# Obturator Hernia in a 71-Year-Old Patient: A Diagnostic Challenge

**DOI:** 10.7759/cureus.42117

**Published:** 2023-07-19

**Authors:** Konstantina Soukouli, Paraskevi Dedopoulou, Athanasios Papatriantafyllou, Ioannis Karioris, Vasileios Leivaditis, Stylianos Tsochatzis

**Affiliations:** 1 Department of Surgery, General Hospital of Patras, Patras, GRC; 2 Department of Cardiothoracic and Vascular Surgery, Westpfalz-Klinikum, Kaiserslautern, DEU

**Keywords:** lower abdominal pain, ct abdomen, bowel obstruction, strangulated hernia, surgical treatments of hernias, obturator hernias

## Abstract

An obturator hernia is a relatively rare form of pelvic hernia, wherein abdominal organs protrude through an opening in the pelvis known as the obturator foramen. The majority of patients with this condition present to the emergency room with symptoms of bowel obstruction. Due to the non-specific nature of these symptoms, making a preoperative diagnosis of obturator hernia can be challenging. Any delay in the diagnosis and treatment of this condition can lead to a significant risk of mortality. In this report, we present the case of a 71-year-old patient who presented to the emergency department complaining of lower abdominal pain and nausea. An abdominal X-ray revealed bowel dilation, and based on the patient’s symptoms, a diagnosis of bowel obstruction was suspected. A CT scan of the abdomen and pelvis was performed to investigate the reason for bowel dilation, and the existence of an obturator hernia was confirmed.

## Introduction

Obturator hernia has an incidence rate of 0.07-1% of all abdominal wall hernias [1]. The first case was documented by De Ronsil in 1724 [2]. This type of hernia primarily affects thin, elderly women, earning it the moniker “the little old lady’s hernia.” Lower abdominal pain is the most prevalent symptom, and most patients exhibit indications of small bowel obstruction. Incarcerated obturator hernias have specific signs, such as obturator neuralgia, Howship-Romberg sign (compression of the obturator nerve), and Hannington-Kiff sign. CT is the preferred diagnostic tool for establishing a diagnosis of obturator hernia before surgery. They typically contain small bowel loops, but can also include other anatomical structures such as the fallopian tube, appendix, omentum, bladder, and ureter.

## Case presentation

On presentation at the emergency department, a 71-year-old female patient complained of pain in the lower left quadrant of the abdomen and nausea that had started 36 hours prior. The patient had an unremarkable medical history. Upon clinical examination, the abdomen was soft with pain located at the left lower abdominal area. No palpable masses or groin masses were detected. In the emergency department, blood pressure was 113/74 mmHg, pulse rate was 69 beats/minute, and temperature was 36.9°C. The laboratory results showed a slightly elevated C-reactive protein.

**Figure 1:**
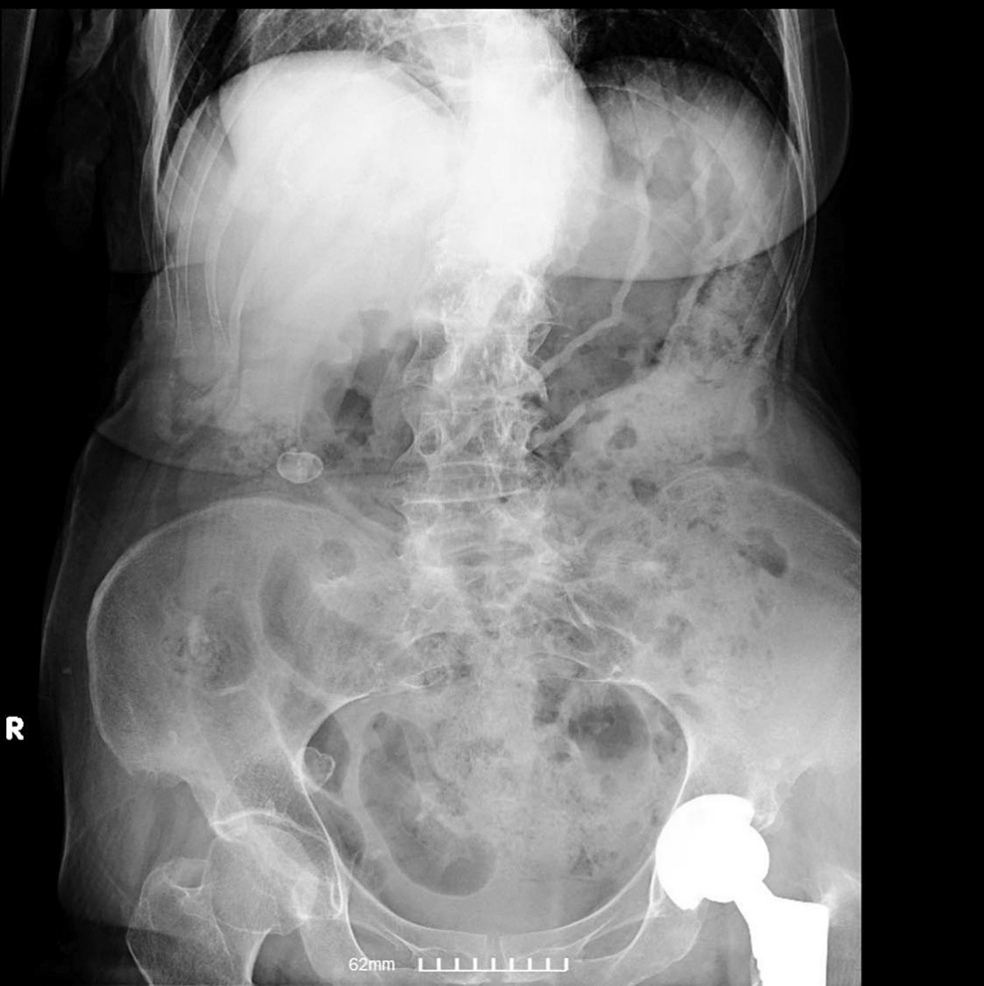
Abdominal X-ray showing dilated loops of bowel.

**Figure 2:**
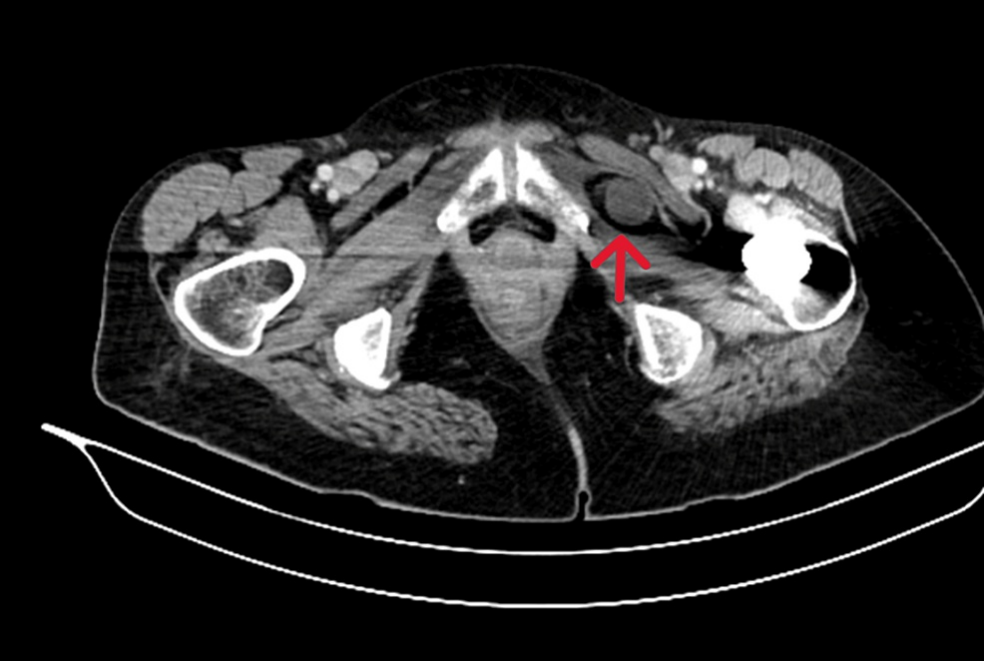
Axial CT scan of the abdomen showing an obturator hernia on the left side (marked by arrow).

**Figure 3:**
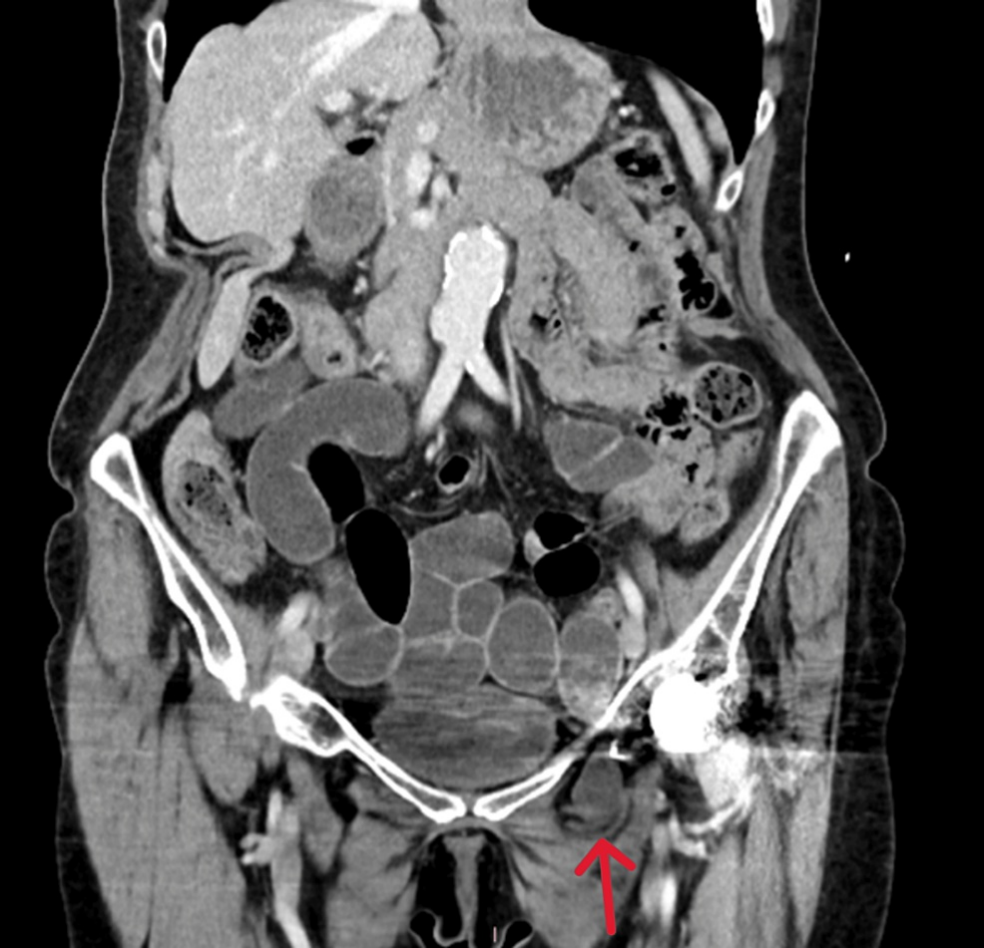
Abdominal coronal CT showing a left-sided obturator hernia (marked by arrow).

An X-ray revealed small bowel obstruction (Figure 1). A CT scan was performed to investigate the cause which confirmed the presence of an obturator hernia (Figures 2, 3).

The patient underwent surgical exploration with a midline incision. During the operation, a loop of small bowel protruding through the obturator canal was identified, which was then reduced back to the abdominal cavity. No signs of bowel ischemia were observed. The defect was repaired with interrupted sutures, and no mesh was used. A drainage was inserted, and the rest of the bowel was carefully checked before closing the abdominal cavity. The patient was discharged six days after admission in stable condition with normal vital signs and no fever.

## Discussion

An obturator hernia is a rare and life-threatening type of hernia that occurs when intra-abdominal organs protrude through the obturator canal, which is penetrated by the obturator neurovascular bundle [3]. This type of hernia is more commonly seen on the right side, as the sigmoid colon usually covers the left obturator foramen. It is most frequently observed in elderly underweight women due to their broader pelvis, low body mass index, and pregnancies; hence, the term “the little old lady’s hernia” [4].

Due to the difficulty of preoperative diagnosis, the mortality rate of obturator hernia is high, ranging from 13% to 40% [5]. Patients typically present with non-specific symptoms such as nausea, lower abdominal pain, and small bowel obstruction, and the hernia is rarely palpable on physical examination. However, there are two helpful specific signs that indicate the presence of an obturator hernia, namely, the Howship-Romberg sign and the Hannington-Kiff sign. The Howship-Romberg sign is characterized by paresthesia and pain in the hip and inner thigh due to pressure on the obturator nerve. Another sign is the Hannington-Kiff sign, where the contraction of the adductor reflex of the adductor muscle is not present after applying pressure to it [3].

Surgery is the only treatment for obturator hernia. The classic surgical approach is through a midline exploratory laparotomy that allows a complete assessment of all abdominal organs, and it is preferred in patients with signs of perforated or strangulated obturator hernias. In hemodynamically stable patients, the laparoscopic approach, which includes the transabdominal preperitoneal (TAPP) and the total extraperitoneal procedures, may also be considered. The TAPP is more appropriate for emergency cases as it allows the exploration of the abdominal cavity. The repair can be performed with simple sutures or with the use of a mesh. The open transabdominal approach remains the gold standard [6,7].

## Conclusions

An obturator hernia is an uncommon type of hernia with the highest mortality rate among all types of hernia. It predominantly occurs in elderly underweight patients. Obturator hernias are difficult to diagnose early, and most patients present with symptoms of small bowel obstruction. In many cases, there is perforation of abdominal viscera, mostly the small bowel. The abdominal CT is considered the diagnostic gold standard. Surgery is the only therapeutic option. Open access is preferred in cases where bowel ischemia is suspected, although the laparoscopic approach is also considered.
